# Size of metastatic deposits affects prognosis in patients undergoing pulmonary metastectomy for colorectal cancer

**DOI:** 10.1308/003588414X13824511650371

**Published:** 2014-01

**Authors:** MA Javed, ARG Sheel, AA Sheikh, RD Page, PS Rooney

**Affiliations:** ^1^Royal Liverpool and Broadgreen University Hospitals NHS Trust,UK; ^2^Liverpool Heart and Chest Hospital NHS Foundation Trust,UK

**Keywords:** Lung neoplasm/secondary, Lung neoplasm/surgery, Colorectal neoplasm/pathology, Pulmonary metastectomy, Survival analysis

## Abstract

**INTRODUCTION:**

Pulmonary metastectomy for colorectal cancer (CRC) is a well accepted procedure although data regarding indications and prognostic outcomes are inconsistent. This study aimed to analyse our experience with resection of pulmonary CRC metastases to evaluate clinically relevant prognostic factors affecting survival.

**METHODS:**

A retrospective analysis was undertaken of the records of all patients with pulmonary metastases from CRC who underwent a thoracotomy between 2004 and 2010 at a single surgical centre.

**RESULTS:**

Sixty-six patients with pulmonary metastases from the colon (*n*=34) and the rectum (*n*=32) were identified. The 30-day hospital mortality rate was 0%, with 63 patients undergoing a R0 resection and 3 having a R1 resection. The median survival was 45 months and the cumulative 3-year survival rate was 61%. Size of pulmonary metastasis and ASA (American Society of Anesthesiologists) grade were statistically significant prognostic factors (*p*=0.047 and *p*=0.009 respectively) with lesions over 20mm associated with a worse prognosis. Sex, age, site, disease free interval (cut-off 36 months), primary tumour stage, hepatic metastases, number of metastases (solitary vs multiple), type of operation (wedge vs lobe resection), hilar lymph node involvement and administration of adjuvant chemotherapy were not found to be statistically significant prognostic factors.

**CONCLUSIONS:**

Pulmonary metastectomy has a potential survival benefit for patients with metastatic CRC. Improved survival even in the presence of hepatic metastases or multiple pulmonary lesions justifies aggressive surgical management in carefully selected patients. In our cohort, size of metastatic deposit was a statistically significant poor prognostic factor.

Colorectal cancer (CRC) is the second leading cause of cancer related mortality in the UK after lung cancer.^1^ Over half of the patients undergoing CRC resection develop recurrence, the most frequent sites being the liver and lung.^2^ Around 10–25% of these patients develop pulmonary metastases and when untreated, pulmonary metastasis has a median survival of less than one year and a five-year survival rate of less than 5%.^3–5^ The first successful excision of pulmonary metastasis was reported by Blalock in 1944.^6^ Subsequently, Thomford *et al* published the principles of surgical resection of lung metastases, a procedure that today has been accepted as a treatment of proven value if the metastatic process is confined to the lungs.^7^

Although pulmonary metastectomy for CRC is a well accepted procedure, reports on indications and prognostic factors affecting survival are inconsistent. Previous studies have reported that five-year survival rates of patients undergoing pulmonary resection range from 24% to 62%, and several prognostic factors including carcinoembryonic antigen (CEA), lymph node status, previous hepatectomy, number of metastases and disease free interval (DFI) have been evaluated.^8^ Rama *et al* have reported a three-year survival rate of 61% in patients undergoing a pulmonary metastectomy for pulmonary metastasis from CRC.^9^

This study aimed to retrospectively analyse our experience of surgical management of patients with pulmonary metastasis from CRC. Primary outcomes were 30-day and 3-year survival. Secondary aims were to examine the prognostic factors affecting overall survival in our cohort of patients.

## Methods

A total of 66 consecutive patients who had undergone pulmonary metastectomy for CRC at the collaborative cardio-thoracic-upper gastrointestinal unit at Liverpool Heart and Chest Hospital between 2004 and 2010 were identified from a prospectively held database. Inclusion criteria were primary site controlled, cardiorespiratory function capable of tolerating complete resection and complete resectability of lung lesions. Patients who had synchronous hepatic metastases had liver surgery prior to pulmonary resection.

All patients were considered for pulmonary metastecto-my on their individual characteristics. Although a short disease free interval (DFI) is recognised as being a poor prognostic indicator, it does not preclude patients from receiving surgery as their therapy if this is thought to be of benefit to them. All patients having pulmonary metastectomy had all their hilar and mediastinal nodal stations sampled. Pathological nodes were resected. A systematic *en bloc* resection of the mediastinum was not part of the surgical strategy in our patients.

Preoperative evaluation of pulmonary nodules was performed using conventional computed tomography (CT). Fludeoxyglucose positron emission tomography (FDG PET) was used if necessary to evaluate suspected metastatic hilar/mediastinal lymph nodes and extrapulmonary disease. All resected specimens were confirmed histopathologically to be pulmonary metastases of CRC.

All patient records were analysed with regard to: age and sex; primary tumour (location, histological differentiation, depth, lymph nodes, lymphatic and venous invasion of the primary tumour); previous hepatectomy for liver metastases; location, size and number of pulmonary metastases; time of appearance of metastases; DFI; use of neoadjuvant and/or adjuvant therapy; type of operation; mortality and follow-up survival. The number of metastatic lesions was evaluated using preoperative CT and intraoperative palpation. When multiple metastases were present, the largest diameter observed was recorded. DFI was calculated from the date of curative surgical treatment of CRC to the date of diagnosis of lung metastases. Survival was calculated from the time of first lung metastectomy to the last date of followup.

The routine follow-up protocol for both primary colorectal resection and pulmonary metastectomy comprised serial chest and abdominal CT obtained every 3–6 months for the first year after surgery, every 6–12 months during 2–5 years after surgery and once a year thereafter. In patients who had a pulmonary metastectomy, serum CEA was measured every 2–3 months in the first year after surgery, every 3–6 months during 2–5 years after surgery and every 6–12 months thereafter. Additional CT was considered when the serum CEA level rose above normal.

### Statistical analysis

Statistical calculations were carried out using StatView^®^ version 5 (SAS Institute, Cary, NC, US). Actuarial survivals were analysed by the Kaplan–Meier method. All variables that revealed a statistically significant difference on univariate analysis were entered into a Cox proportional hazards regression model for multivariate analysis. A *p*-value of <0.05 was considered statistically significant.

## Results

The median age at pulmonary resection was 67 years (range: 55–79 years). There were 21 men and 45 women in our cohort. Sixty-three patients (95.5%) underwent a R0 resection and there were three R1 resections. The median DFI was 19.5 months (range: 13–93 months). The median duration between date of diagnosis and surgery was 9.5 weeks (interquartile range: 3–41 weeks). The primary colorectal tumour was located in the colon in 34 patients and in the rectum in 32.

Eight patients received pulmonary neoadjuvant chemotherapy and twenty-three had adjuvant chemotherapy. Chemotherapy was given to patients depending on the perceived benefits based on their individual characteristics and their fitness to withstand treatment. Twenty-six patients (39.3%) had developed liver metastases and underwent a complete hepatic metastectomy before the thoracotomy. Forty-two patients (63.6%) had a solitary lesion and 24 (36.3%) had multiple lesions. Of the latter, 15 patients had unilateral deposits and 9 had bilateral metastasis. In 48 patients, the DFI was less than 36 months, and the remaining 18 had a DFI of >36 months.

Wedge resection or segmental resection was performed in 47 patients, lobectomy in 17 and pneumonectomy in 3. Pneumonectomies were carried out to obtain complete macroscopic clearance of the disease. Although we would prefer to carry out sublobar resections, lobectomies or sleeve lobectomies, this was not possible on these three occasions. All patients with bilateral disease had staged thoracotomies.

**Figure 1:**
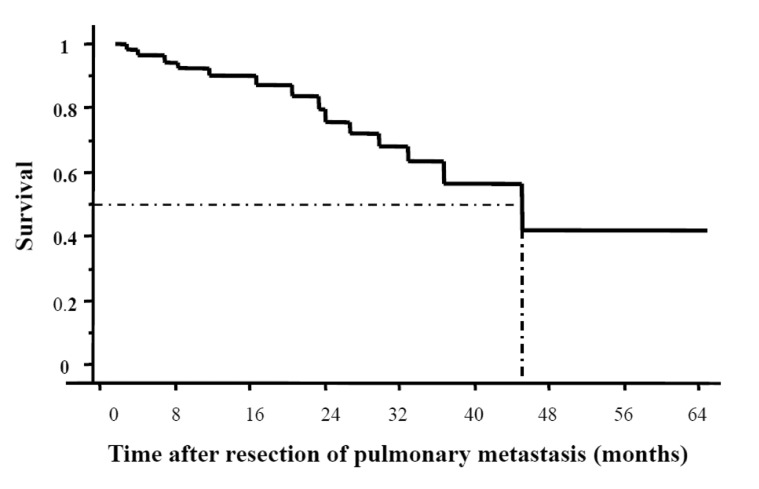
Kaplan–Meier curve showing overall 3-year survival rate of 61% and median survival of 45 months

There was no in-hospital mortality or 30-day mortality in our cohort. The median survival was 45 months and the cumulative 3-year survival rate was 61% (Fig 1). Univariate analysis revealed that ASA (American Society of Anesthesiologists) grade and size of pulmonary tumours represented significant prognostic factors for overall survival (Table 1). DFI (36 months), site or differentiation of primary tumour, previous hepatic metastasis, number of pulmonary metastases, pulmonary adjuvant or neoadjuvant therapy, type of resection and hilar lymph node involvement had no effect on survival. In multivariate analysis, ASA grade and size of the lesion were again identified as independent prognostic factors (Table 2).

**Table 1:** Survival and univariate analysis of prognostic factors of patients who underwent curative metastatectomy

Variables	Categories	Cases (*n*=66)	Median survival	Univariate analysis *p*-value
Age	<60 years	26	45 months	0.50
	≥60 years	40	38 months	
ASA grade	1	20	67 months	**0.009**
	2	46	33 months	
Sex	Female	45	41 months	0.07
	Male	21	37 months	
Primary location	Colon	34	36 months	0.25
	Rectum	32	40 months	
Neoadjuvant therapy	Yes	20	43 months	0.69
	No	46	39 months	
Hepatic metastasis	Yes	26	33 months	0.86
	No	40	43 months	
Disease free interval	<36months	48	43 months	0.53
	>36months	18	45 months	
Size of metastatic deposit	<20mm	31	46 months	**0.0047**
	>20mm	35	24 months	
Number of metastases	Single	42	33 months	0.55
	Multiple	24	36 months	
Distribution of metastasis	Unilateral	56	37 months	0.46
	Bilateral	10	33 months	
Vascular invasion	Yes	3	25 months	0.28
	No	63	36 months	
Pathological involvement of hilar and/or	Yes	4	29 months	0.90
mediastinal nodes	No	62	43 months	
Type of resection	Wedge/segmental	47	33 months	0.42
	Lobectomy	17	41 months	
	Pneumonectomy	3	40 months	
Pulmonary neoadjuvant chemotherapy	Yes	8	34 months	0.76
	No	58	43 months	
Pulmonary adjuvant chemotherapy	Yes	23	36 months	0.50
	No	43	44 months	
Dukes’ stage of primary tumour	A and B	24	40 months	0.67
	C and D	42	31 months	

ASA = American Society of Anesthesiologists

**Table 2:** Multivariate analysis of prognostic factors for survival

	Hazard ratio (95% confidence interval)	*p*-value
**Size of lesion**<20mm>20mm	0.334 (0.116–0.936)	**0.043**
**ASA grade**12	0.553 (0.118–0.935)	**0.029**

**Figure 2:**
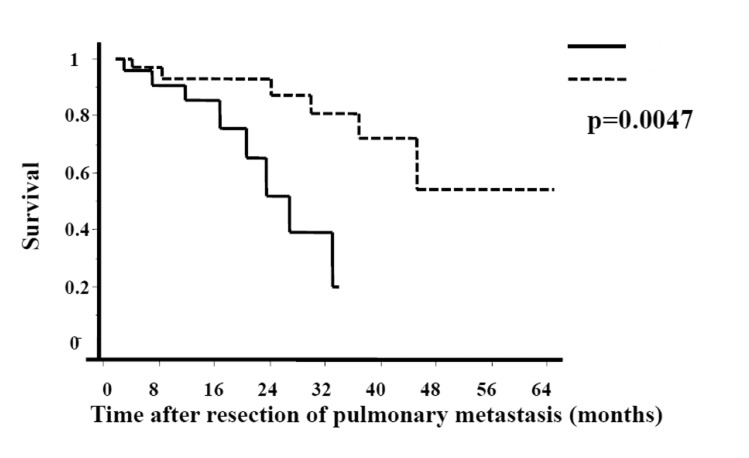
Kaplan–Meier curves showing overall survival after curative resection of pulmonary metastasis. Patients grouped according to size of pulmonary deposit

**Figure 3:**
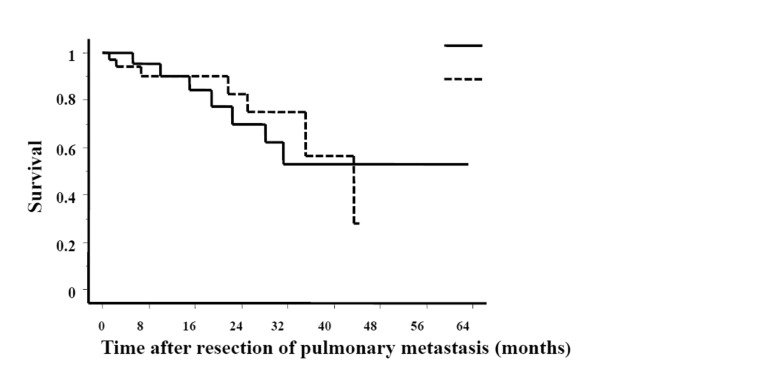
Kaplan–Meier curves showing overall survival after curative resection of pulmonary metastasis. Patients grouped according to presence or absence of concurrent hepatic metastasis

The median survival of patients in whom size of pulmonary metastasis was <20mm was approximately 47 months compared with 25 months in those with pulmonary deposits of >20mm. Figure 2 compares the Kaplan–Meier survival curves of patients in the above two groups demonstrating a significant difference in survival (*p*=0.0047). However, there was no significant difference in the median survival of patients who had undergone hepatic metastectomy (33 months) compared with those who had no history of surgery for liver metastasis (43 months) (Fig 3).

## Discussion

Pulmonary metastectomy in CRC is a well recognised procedure and has been proven to improve survival.^2,8^ Our study, which is the largest reported series from the UK, demonstrates that long-term survival can be expected after resection of pulmonary metastasis from CRC, particularly in patients with metastasis of <20mm. The overall three-year survival rate in this study was 61%, which compares very well with survival rates reported previously.^9–11^ The fact that there was no in-hospital or 30-day mortality also highlights the effectiveness of centralisation of services in high volume specialised centres.

In previous studies, size of pulmonary deposit was not found to be a significant prognostic parameter.^9,11–14^ Conversely, our results demonstrate – for the first time – that size of pulmonary metastasis (cut-off 20mm) is an independent prognostic factor for survival in patients undergoing a metastectomy for CRC. The cut-off of 20mm was chosen as this was the median size of metastatic deposit in our series. Unsurprisingly, the outcome of patients with ASA grade 1 was better than for those with ASA grade 2.

Studies have shown that resection of both hepatic and pulmonary metastases secondary to CRC in selected patients is safe and results in long-term survival.^15–19^ In our series, 47% of patients with a primary colonic tumour (16 of 34) and 31% of patients with primary rectal cancer (10 of 32) had liver metastasis. Survival of the 26 patients who had a history of hepatic metastectomy was not statistically different to those without liver metastasis. These findings justify pulmonary resection in patients with previously resected liver metastasis provided that the performance status of the patients is good.

Various other prognostic indicators for patients who undergo a thoracotomy for lung metastases from CRC have been examined in previous studies. As indicated by others,^20–23^ neither age nor sex nor the location of the primary carcinoma (colon or rectum) influenced prognosis in our series. However, reports regarding the prognostic significance of other factors such as DFI, number and distribution of pulmonary metastases, chemotherapy, lymph node/vascular invasion and stage of primary tumour are inconsistent^8–13,23–25^ and none of these were statistically significant in our analysis.

### Study limitations

This study has some limitations. Although prethoracotomy serum CEA is considered a prognostic factor in patients undergoing pulmonary resection from CRC,^19,21–23,26^ routine CEA estimation was not part of the CRC follow-up regime in our patients (since there was no national consensus in this group of patients during the study period) and routine imaging at one and two years picked up most patients with pulmonary metastasis. The lack of a non-surgical group in our cohort also makes it difficult to make a comparison. A multicentre retrospective study in metastatic CRC patients from 2012, however, revealed a significantly longer progression free and overall survival in patients undergoing a pulmonary metastectomy than those who received chemotherapy alone.^27^

Since our study was not randomised, some bias in terms of preoperative assessment or chemotherapy may have been present as well, contributing to patient selection bias. It is, nevertheless, important to mention that the first randomised controlled trial comparing the outcome of patients randomly allocated ‘active monitoring’ versus ‘active monitoring with pulmonary metastectomy’ is underway (Pulmonary Metastasectomy in Colorectal Cancer [PulMiCC]) and the findings from this trial would aid physicians and surgeons to optimise management of patients with pulmonary metastasis from CRC.^28^

## Conclusions

Our experience indicates that pulmonary metastectomy carries a potential survival benefit for patients with metastatic colorectal carcinoma. Low morbidity and mortality rates in carefully selected patients justify the aggressive approach of surgical management. We report that size of pulmonary deposits is a significant independent prognostic factor for medium-term survival.
